# Complete Genome Sequences of *Streptomyces* Bacteriophages Annihilus, TonyStarch, Thiqqums, CricKo, ClubPenguin, RosaAsantewaa, and PherryCruz

**DOI:** 10.1128/mra.00922-22

**Published:** 2022-10-26

**Authors:** Yvette Genie Park, Gillian Faith McCarthy, Hadeeqa Mustafa, Gemma M. Feild, Snigdha Puram, Hager Aly Younes, Danyah Imam, 2021 UMBC Phage Hunters, STEM BUILD at UMBC Cohort 5, Ivan Erill, Steven M. Caruso

**Affiliations:** a Department of Biological Sciences, University of Maryland Baltimore County, Baltimore, Maryland, USA; Queens College CUNY

## Abstract

Seven siphoviruses were isolated from soil using *Streptomyces* hosts. Their genome sequences ranged from 42,730 to 57,624 bp long and had a GC content of approximately 60%. Based on their gene content similarity to actinobacteriophages, all seven phages were assigned to cluster BI. For several of these phages, multiple ribosomal frameshifts were identified.

## ANNOUNCEMENT

**FIG 1:**
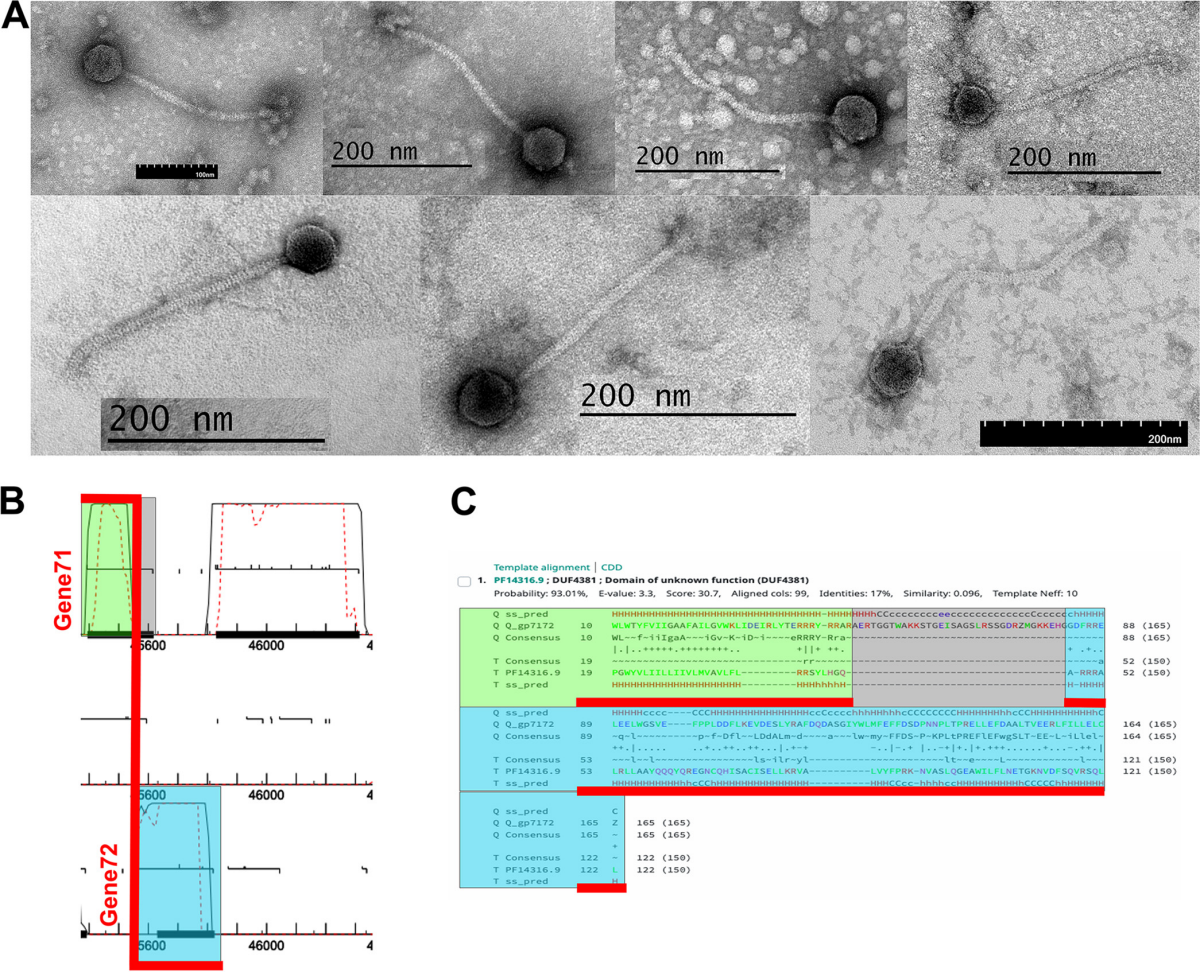
Virion imaging and putative frameshift identification in cluster BI phages. (A) Representative transmission electron microscopy images of the phages described in this paper. (Left to right, top row) Annihilus, ClubPenguin, CricKo, and PherryCruz; (bottom) RosaAsantewaa, Thiqqums, and TonyStarch. (B) Annotated GeneMark coding potential for TonyStarch genes 71 and 72. Green and blue highlights indicate the coding potential for genes 71 and 72 involved in a hypothetical ribosomal frameshift. Gray highlight indicates the coding potential of gene 71 not included in that ribosomal frameshift. (C) HHpred was used to identify a conserved domain (PF14316) using a literal concatenation of gene product 71 and 72 hits as the query, but the coding potential of gene 71 not included in the ribosomal frameshift was not required for the alignment. Using the predicted frameshift sequence as the query resulted in an improved alignment score (35) and probability (95.85%). Color coding as in panel B.

*Streptomyces* species are well known for the production of antibiotics and other bioactive compounds. Here, we report on seven bacteriophages isolated from soil samples on two members of the genus, Streptomyces scabiei RL-34 (ATCC 49173), a plant pathogen that causes potato scab disease (1), and Streptomyces mirabilis NRRL-2400 (ARS), a species able to grow in soils containing heavy metals (2), using standard methods (3) (Table 1). Briefly, soil samples were washed in phage buffer (10 mM Tris [pH 7.5], 10 mM MgSO_4_, 1 mM CaCl_2_, 68.5 mM NaCl), and the wash was collected by centrifugation and filtration (0.22-μm filter). The filtrate was then plated in tryptic soy soft agar (BD), with either *S. scabiei* or *S. mirabilis* overlaid on nutrient agar (BD Difco) supplemented with 10 mM MgCl_2_, 8 mM Ca(NO_3_)_2_, and 0.5% glucose, and incubated at 30°C for 1 to 2 days to yield bacteriophages Annihilus, TonyStarch, Thiqqums, CricKo, ClubPenguin, and RosaAsantewaa. For one soil sample, the filtrate was first inoculated with *S. scabiei* RL-34 and incubated with shaking for 24 h at 30°C; then, the culture was filtered and plated in soft agar with *S. scabiei* yielding phage PherryCruz. All phages were purified with a minimum of three rounds of plating. Negative stain transmission electron microscopy revealed all seven bacteriophages to be siphoviruses (4) (Fig. 1A). The particle capsid and tail measurements are provided in Table 1.

**TABLE 1:** Summary properties of the analyzed BI *Streptomyces* bacteriophages

Phage name	Collection location	Host	Particle head diam ± SD, tail length ± SD (nm); no. of particles	Total no. of 150-bp reads	Cluster	Genome length (bp)	GC content (%)	3′ single-stranded end sequence	GenBank accession no.	SRA accession no.
TonyStarch	Ellicott City, MD (39.262 N, 76.818 W)	*S. mirabilis*	55 ± 1, 285 ± 8; 3	972,262	BI1	55,469	59.6	5′-CGCCCGCCT-3′	ON108646	SRX14485098
Annihilus	Glen Burnie, MD (39.133021 N, 76.628615 W)	*S. scabiei*	52 ± 2, 238 ± 24; 4	1,895,132	BI2	43,562	61.2	5′-CGCCGCCCT-3′	ON081336	SRX14443513
PherryCruz	Halethorpe, MD (39.257417 N, 76.704278 W)	*S. scabiei*	52 ± 2, 228 ± 8; 8	1,005,742	BI2	43,736	61.0	5′-CGCCGCCCT-3′	MK686070	SRX14814648
RosaAsantewaa	Accra, Ghana (5.6052560 N, 0.1733080 W)	*S. mirabilis*	47 ± 4, 234 ± 15; 5	832,774	BI2	42,730	58.8	5′-CGCCCGCCT-3′	MK686072	SRX14814650
CricKo	Baltimore, MD (39.25366 N, 76.71331 W)	*S. scabiei*	62 ± 3, 271 ± 6; 4	1,086,915	BI4	57,623	58.1	5′-CGCCCGCCT-3′	MT310854	SRX14814644
Thiqqums	Catonsville, MD (39.2585 N, 76.7131 W)	*S. scabiei*	50, 200; 1	932,673	BI4	57,624	58.1	5′-CGCCCGCCT-3′	MT657340	SRX14814651
ClubPenguin	Millersville, MD (39.096152 N, 76.573399 W)	*S. mirabilis*	61 ± 2, 264 ± 16; 5	913,162	BI7	56,205	59.0	5′-CGCCCGCCT-3′	MT310852	SRX14814643

Genomic DNA of all seven bacteriophages was isolated from crude lysate and purified using a Promega Wizard DNA cleanup system, prepared for sequencing using the NEB Ultra II library kit, and sequenced at the Pittsburgh Bacteriophage Institute using the Illumina MiSeq platform (v3 reagents), producing over 100,000 150-base single-end reads for each phage (Table 1). The raw reads were assembled using Newbler v2.9. Quality control was performed using Consed v29 (5). The genome ends were identified by comparison to similar phages with known ends and confirmed by read start buildups. Based on the gene content similarity, all seven phages were assigned using PhagesDB to actinobacteriophage cluster BI (6–8). The sequencing data, genome characteristics, and cluster assignments are provided in Table 1.

Genome annotation was completed using DNA Master v5.23.6 (9) embedded with Glimmer v3.02b (10), GeneMark v4.28 (11), Phamerator v.Actino_draft 463 (12), NCBI blastp v2.13.0 (13), and HHpred v57c87 (14). The phages were found to have from 55 (RosaAsantewaa) to 94 (CricKo, Thiqqums) protein coding genes, of which an average 32% were assigned functions. No tRNA coding genes were identified using tRNAscan-SE v2.0 (15) or Aragorn v1.2.41 (16).

In the BI1 and BI2 phages examined here, the heuristic GeneMarkS algorithm (11) was used to predict multiple programmed ribosomal frameshifts. In TonyStarch (BI1), most putative frameshifts were in genes located near predicted endolysins and nucleases, while in the cluster BI2 phages (Annihilus, PherryCruz, RosaAsantewaa), they were in genes near predicted holins. Some of these putative frameshifts may be functional, since the predicted products for frameshifts involving TonyStarch genes 4 to 5 and 71 to 72 improved the HHpred alignment to known protein domains compared to the literal concatenation of the respective gene products (Fig. 1B and C).

### Data availability.

The GenBank accession numbers for the genome sequences reported here and the SRA accession numbers for the raw sequence reads are available in Table 1.
